# Exploring the Biomechanical Properties of the Human Cornea *In Vivo* Based on Corvis ST

**DOI:** 10.3389/fbioe.2021.771763

**Published:** 2021-11-17

**Authors:** Di Zhang, Haixia Zhang, Lei Tian, Yan Zheng, Caiyun Fu, Changbin Zhai, Lin Li

**Affiliations:** ^1^ Beijing Key Laboratory of Fundamental Research on Biomechanics in Clinical Application, Capital Medical University, Beijing, China; ^2^ School of Biomedical Engineering, Capital Medical University, Beijing, China; ^3^ Beijing Tongren Eye Center, Beijing Tongren Hospital, Beijing Ophthalmology and Visual Sciences Key Laboratory, Beijing Institute of Ophthalmology, Capital Medical University, Beijing, China; ^4^ Beijing Advanced Innovation Center for Big Data-Based Precision Medicine, Beijing Tongren Hospital, Beihang University and Capital Medical University, Beijing, China; ^5^ Beijing Tongren Eye Center, Beijing Tongren Hospital, Beijing Ophthalmology and Visual Sciences Key Laboratory, Capital Medical University, Beijing, China

**Keywords:** cornea, corneal biomechanical properties, *in vivo*, CorVis ST, visco-hyperelastic model

## Abstract

**Purpose:** The aim of this study was to provide a method to determine corneal nonlinear viscoelastic properties based on the output data of corneal visualization Scheimpflug technology (Corvis ST).

**Methods:** The Corvis ST data from 18 eyes of 12 healthy humans were collected. Based on the air-puff pressure and the corneal displacement from the Corvis ST test of normal human eyes, the work done by the air-puff attaining the whole corneal displacement was obtained. By applying a visco-hyperelastic strain energy density function of the cornea, in which the first-order Prony relaxation function and the first-order Ogden strain energy were employed, the corneal strain energy during the Corvis ST test was calculated. Then the work done by the air-puff attaining the whole corneal displacement was completely regarded as the strain energy of the cornea. The identification of the nonlinear viscoelastic parameters was carried out by optimizing the sum of difference squares of the work and the strain energy using the genetic algorithm.

**Results:** The visco-hyperelastic model gave a good fit to the data of corneal strain energy with time during the Corvis ST test (*R* ^2^ > 0.95). The determined Ogden model parameter *μ* ranged from 0.42 to 0.74 MPa, and *α* ranged from 32.76 to 55.63. The parameters *A* and *τ* in the first-order Prony function were 0.09–0.36 and 1.21–1.95 ms, respectively.

**Conclusion:** It is feasible to determine the corneal nonlinear viscoelastic properties based on the corneal contour information and air-puff pressure of the Corvis ST test.

## Introduction

Cornea is a kind of biological soft tissue with nonlinear viscoelasticity. The biomechanical property of cornea has been considered as a helpful index in the diagnosis of keratoconus (Wang et al., 2015), selection of refractive surgery (Dupps and Wilson, 2006; Shen et al., 2014), screening before refractive surgery (Piñero and Alcón, 2014), measurement of intraocular pressure (IOP) (Brown et al., 2018), and evaluation of the curative effect of corneal cross-linking surgery (Vinciguerra et al., 2017; Sedaghat et al., 2018).

At present, corneal visualization Scheimpflug technology (Corvis ST) has been used in the diagnosis of keratoconus (Chan et al., 2018) and glaucoma (Wang et al., 2015). Corneal viscoelasticity is closely related to the progressive keratoconus (Fraldi et al., 2011; Kling et al., 2014). However, the mechanical meaning of the obtained dynamic corneal response (DCR) parameter output by Corvis ST has not been clear exactly. The relationship between DCR parameters and classical biomechanical properties has not been established either. More importantly, the DCR parameters from Corvis ST could not be directly used by numerical methods, such as finite element methods, to predict corneal deformation after refractive surgery. Therefore, many researchers have paid great attention to attempt to have a comprehensive understanding of corneal biomechanical properties based on the tests of cornea *in vivo*. By applying inverse finite element method and corneal deformation data from Corvis ST, investigators studied the nonlinear mechanical characteristics of the cornea (Lago et al., 2015; Sinha Roy et al., 2015; Bekesi et al., 2016), in which a complicated constitutive equation and a fine geometry model were applied. Our previous study (Qin et al., 2019a) applied a thin spherical shell model to identify corneal elastic modulus based on Corvis ST data. These studies have demonstrated the feasibility of using the output data of Corvis ST to acquire corneal biomechanical parameters, such as elastic modulus. However, a full description of corneal biomechanical properties based on *in vivo* tests has been still an issue of great concern to the researchers.

This study will establish a method to identify corneal nonlinear viscoelastic mechanical properties based on the output data of Corvis ST. We shall apply a visco-hyperelastic model to fit the corneal deformation data extracted from the image information during a Corvis ST test. It is expected that the method of determination of corneal nonlinear viscoelastic parameters can be used in the optimization design of corneal refractive surgery scheme and the prediction of corneal morphology after refractive surgery.

## Materials and methods

### Subjects

This study included 18 eyes of 12 healthy subjects. The institutional review board of Capital Medical University approved this study, and all participants signed an informed consent form in accordance with the tenets of the Declaration of Helsinki.

Subjects met the following criteria: age 18–45 years. All patients had no history of corneal, ocular surgery, trauma, or systemic diseases that might affect the eye and had abandoned soft contact lenses or rigid contact lenses at least 1 month and no contact lens utilization within 2 weeks before the examination.

### Ocular Examination

All subjects underwent a thorough tomographic measurement using Corvis ST (Oculus; Optikgeräte GmbH, Wetzlar, Germany, software version: 1.6b2042) and Pentacam (Oculus; Optikgeräte GmbH, Wetzlar, Germany). The DCR parameters in the analysis mainly included deflection amplitude at the highest concavity (HCDA), central concave curvature at the highest concavity (HCR), peak distance at the highest concavity (PD), deflection amplitude ratio maximal 2 mm (DAR2), stiffness parameter at the first applanation (SPA1), corneal stress–strain index (SSI), corneal biomechanical index (CBI), and biomechanically corrected IOP (bIOP).

### Method to Evaluate Corneal Nonlinear Viscoelasticity

#### Visco-Hyperelastic Model

Considering the time dependence of corneal stress responding to any loads, a visco-hyperelastic model based on strain energy function has been shown to describe corneal nonlinear and viscoelastic properties (Liu et al., 2020a; Su et al., 2015; Whitford et al., 2018). As one of well-known isotropic strain energy functions, the Ogden model can better describe the nonlinear stress–strain relationship of the cornea (Bao et al., 2017; Bao et al., 2018; Joda et al., 2016; Lago et al., 2015; Maklad et al., 2019). Therefore, we adopted the Ogden model in the visco-hyperelastic constitutive model. Also, the widely used Prony series model (Huang et al., 2017; Ramzanpour et al., 2020) as relaxation function was introduced in the visco-hyperelastic model. The cornea was regarded as a nonlinear incompressible viscoelastic material. The visco-hyperelastic model (Snedeker et al., 2005; Taylor et al., 2009) (Eq. 1) was applied, in which the strain energy density *U* was given by the Ogden model (Eq. 2), and the stress relaxation function *G* was given by the first-order Prony model (Eq. 3).

$$U\_e=\overset{t}{\underset{0}{\int }}G\left(t-s\right)\frac{\partial U}{\partial s}ds$$ (1)

$$U=\overset{N}{\underset{i=1}{\sum }}\frac{2\mu_{i}}{a_{i}^{2}}\left(\lambda_{1}^{a_{i}}+\lambda_{2}^{a_{i}}+\lambda_{3}^{a_{i}}-3\right),\lambda_{1}\lambda_{2}\lambda_{3}=1$$ (2)

$$G\left(t\right)=1-A\left(1-e^{-\frac{t}{\tau }}\right)$$ (3)

In Eq. 2, *N*, *μ*_*i*_, and *α*_*i*_ are material parameters, and *λ*_*j*_ (*j* = 1, 2, 3) is the stretch ratio of materials in three main directions. Taking into consideration that the first-order Ogden model can better describe the mechanical properties of cornea (Bao et al., 2017; Bao et al., 2018), in this study, we let *N* = 1. In Eq. 3, *A* and *τ* are the stress relaxation parameters, 1−A is the relaxation limit, and *τ* is the relaxation time.

It is assumed that the three main directions of cornea are circumferential, radial, and perpendicular (or thick) directions. It is further assumed that the circumferential elongation ratio is equal to the elongation ratio of the radial arc length under air-puff. It follows that $\lambda_{1}=\lambda_{2}=\lambda$ , $\lambda_{3}=\lambda^{-2}$ due to the assumption of incompressibility of the cornea, i.e., $\lambda_{1}\lambda_{2}\lambda_{3}=1$ . Then, Eq. 2 gives

$$U=\frac{2\mu }{\alpha^{2}}\left(2\lambda^{\alpha }+\lambda^{-2\alpha }-3\right)$$ (2a)

According to the corneal contour imagines obtained from Corvis ST, the overall corneal strain under the air-puff at each time point was calculated. The stretch ratio was set by Eq. 4.

$$\lambda =\frac{L_{1}}{L_{0}}$$ (4)

where *L*_1_ and *L*_0_ are the arc length of the anterior corneal surface during deformation and at the initial state, respectively. *L*_1_ and *L*_0_ can be obtained from the corneal anterior surface contour by image processing.

#### The Work Done by Air-Puff During the Corvis ST Test

**FIGURE 1:**
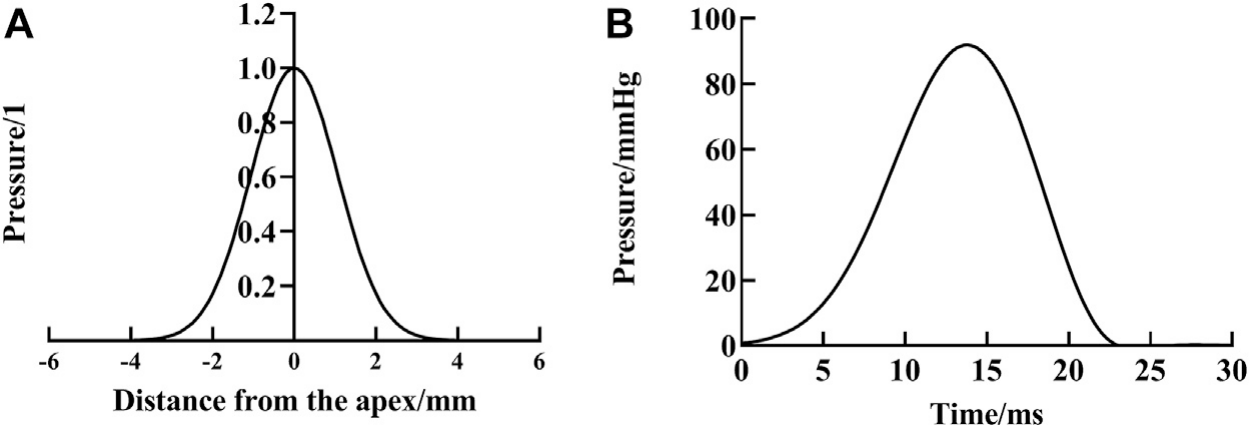
Air-puff applied to cornea by Corvis ST, variation of space **(A)** and time **(B)**.

**FIGURE 2:**
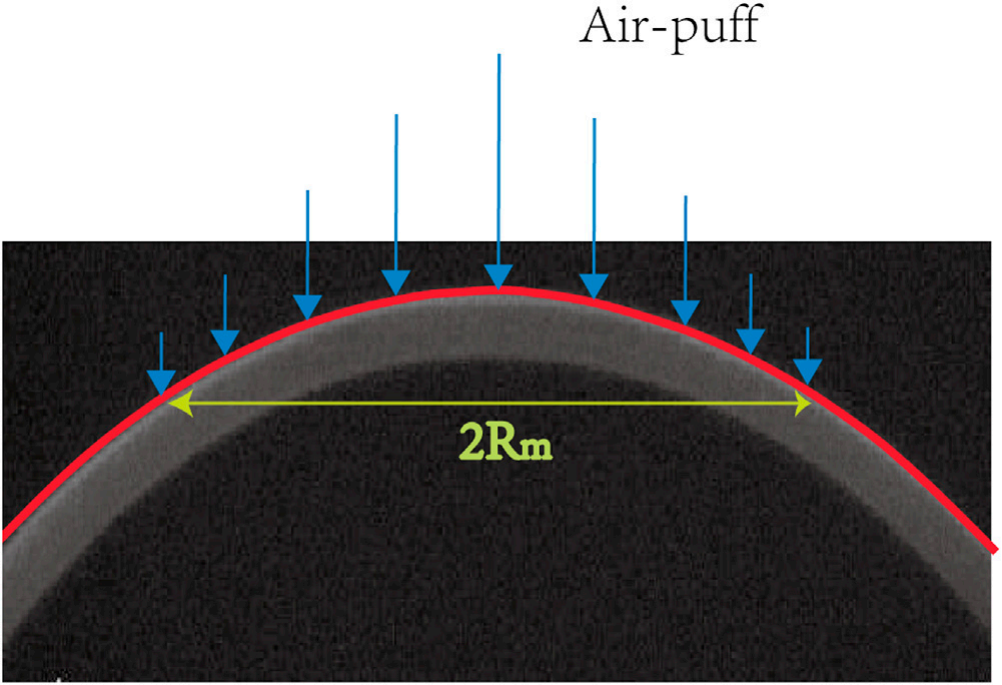
Schematic of air-puff acts on the cornea during the Corvis ST test.

We employed cylindrical coordinates to describe the work done by air-puff attaining the whole corneal displacement during the Corvis ST test. According to literatures (Simonini et al., 2016; Eliasy et al., 2019), the pressure of the air-puff, *P*(*r*, *t*), at the point with polar coordinate *r* from the corneal apex and time *t* was obtained (Figure 1). We let *S*(*r*, *t*) be the corneal vertical displacement of the point *r* from the corneal apex on the anterior surface of the cornea at time *t.* Then for an element rdrdθ on the corneal anterior surface, the work element was given by dw=P(r,t)S(r,t)rdrdθ . The work done by the air-puff attaining the whole corneal displacement up to time *t* from the beginning of a Corvis ST test can be obtained according to Eq. 5.

$$W_{e}=\int_{0}^{2\pi }\int_{0}^{R_{m}}P\left(r,t\right)S\left(r,t\right)rdrd\theta =2\pi \int_{0}^{R_{m}}P\left(r,t\right)S\left(r,t\right)rdr$$ (5)

where *R*_*m*_ is the radius of the maximum deformed area of the corneal anterior surface by the air-puff; *R*_m_ can be estimated by the data of the corneal anterior surface contour (Figure 2).

#### The Deformation Data of the Cornea During the Corvis ST Test

**FIGURE 3:**
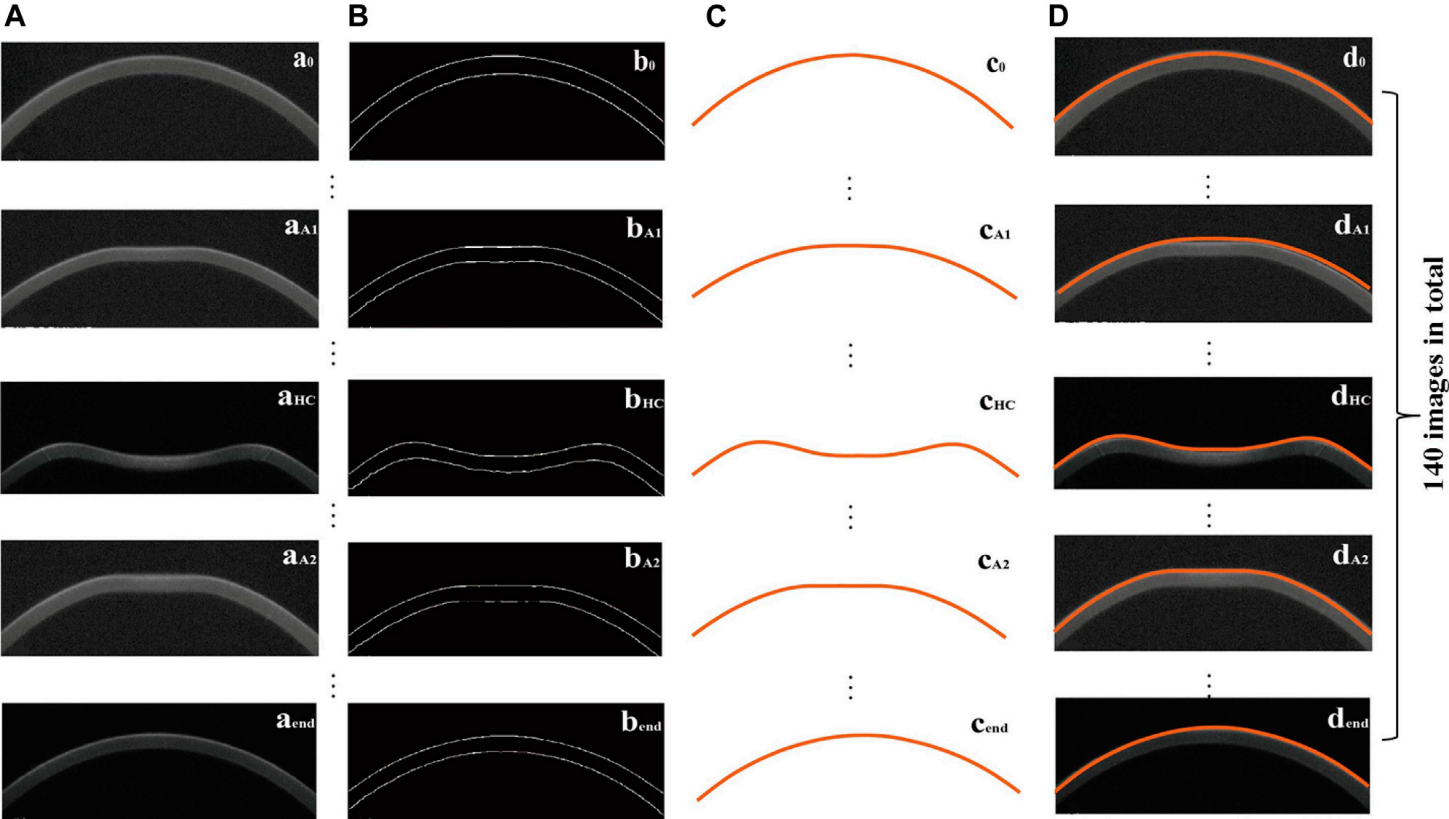
Original corneal image (a_n_) **(A)**, the corresponding processed image (b_n_) **(B)**, extracted corneal anterior surface contour (c_n_) **(C)**, and the position of the extracted corneal anterior surface contour in the original image (d_n_) **(D)**, where *n* = 0 (initial state), A_1_ (first applanation), HC (highest concavity), A_2_ (second applanation), end.

**FIGURE 4:**
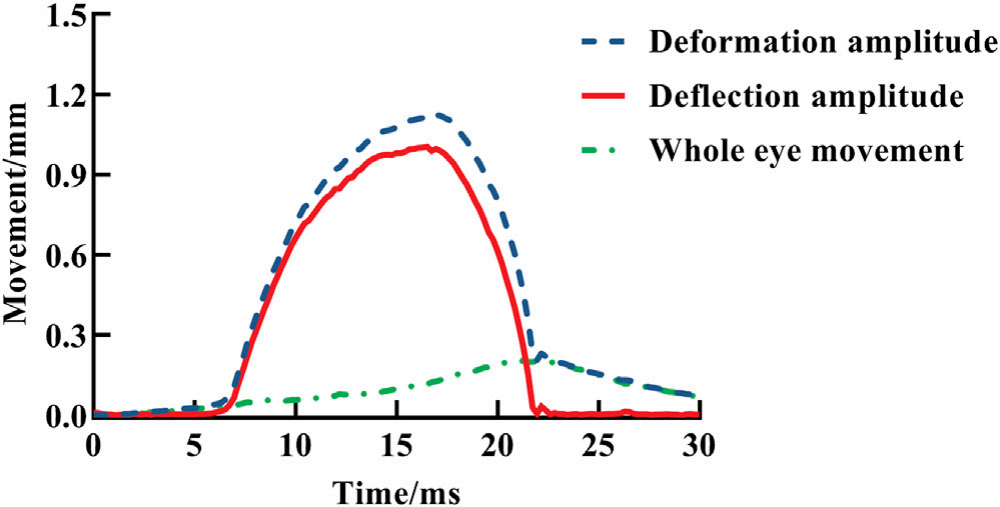
Change of apex total displacement, apex pure displacement, and eye globe movement with time, indicated by blue dotted line, red solid line, and green dotted line, respectively.

In this study, according to the corneal contour images obtained from Corvis ST, we extracted the outer contour of the cornea (Figure 3) according to the OTSU threshold method. *L*_1_ and *L*_0_ in Eq. 4 were obtained from the extracted corneal anterior surface contour, where $L_{1}=\overset{286}{\underset{i=-287}{\sum }}\sqrt{{\left(x_{i+1}-x_{i}\right)}^{2}+{\left(y_{i+1}-y_{i}\right)}^{2}}$ ; (*x*_*i*_, *y*_*i*_) is the coordinate of a point on the anterior surface of the cornea, where the *Y* axis passes through the corneal apex in the Cartesian coordinate system. Moreover, the difference between the initial corneal contour (*t* = 0) and the outer contour of the cornea at time *t* was recorded as vertical displacement of anterior corneal surface at the time *t.* Then, the vertical displacement of the anterior corneal surface during the Corvis ST test was obtained after removing the whole eye movement (the displacement of the whole eyeball is shown in Figure 4).

In order to complete the calculation of the work done by air-puff attaining the whole corneal displacement based on Eq. 5, we need to fit these deformation data sets. We applied

$$S\left(r,t\right)=\left(w_{0}-w_{1}e^{S_{1}\left(t-c\right)-S_{2}{\left(t-c\right)}^{2}-S_{3}{\left(t-c\right)}^{3}-S_{4}{\left(t-c\right)}^{4}}\right)\left(a_{1}e^{-{\left(\frac{r-b_{1}}{c_{1}}\right)}^{2}}+a_{2}e^{-{\left(\frac{r-b_{2}}{c_{2}}\right)}^{2}}\right)$$ (6)

to fit the vertical displacement of the anterior surface of the cornea at point (*r*, *t*), where 0 ≤ *t* ≤ 30 (ms), 0 ≤ *r* ≤ *R*_m_ (mm). According to the extracted corneal anterior surface contour, the radius of the maximum corneal deformation area is 4.29 mm, i.e., *R*_m_ is 4.29 mm. Parameter *c* is the time when the corneal apex in the corneal vertical displacement time curve is at the maximum position which is given from Corvis ST data.

#### The Determination of the Material Parameters

The cornea was regarded as a nonlinear incompressible viscoelastic material, and the Corvis ST test process was assumed to be a loading and unloading process with constant strain rate. The work done by an air-puff attaining the whole corneal displacement was completely regarded as the strain energy. Based on Eqs. 1, 2a, 3–6, the material parameters could be determined by minimizing the objective function shown in Eq. 7.

$$\mathrm{RM}=\sum_{k=1}^{n}{\left(W_{ek}-VU_{k}\right)}^{2}$$ (7)

where $W_{ek}\;$ is the work done by the air-puff attaining the whole corneal displacement during the Corvis ST test which is computed according to Eq. 5, and $U_{k}\;$ is the strain energy density theoretical value which is calculated by Eq. 1. The *n* in Eq. 7 is the number of the selected time points within 30 ms (The time of 30 ms was evenly divided into 71 time periods). *V* is the volume of the cornea in a zone around the anterior corneal apex with an 8.5-mm diameter. In this study, *n* was set 71, and *V* was from the Pentacam data.

The material parameters needed to identify were μ, α,A , and τ . The genetic algorithm based on Matlab (R2020b, MathWorks, Natick, MA, USA) was applied to solve the minimization of *RM* in Eq. 7. The value ranges of material parameters were set according to the literatures (Lago et al., 2015; Bao et al., 2018; Qin et al., 2019b).

### Statistical analysis

All analyses were performed using SPSS (version 23.0, IBM Corporation, Armonk, NY, USA). The data were tested for normality of distribution using the Shapiro–Wilk test and expressed as mean ± SD or median (interquartile range, IQR). Power of the tests was calculated using the data of the determined parameter *μ.*

## Results

This study included 18 eyes of 12 participants (9 males, 3 females); the baseline information of participants is shown in Table 1.

**TABLE 1:** Baseline information of the human eyes.

Parameters	Age (years)	CCT (μm)	bIOP (mmHg)
Mean ± SD	23.4 ± 2.9	573 ± 24	16.06 ± 2.37

CCT: central corneal thickness.

### DCR parameters from Corvis ST

The values of DCR parameters in the eyes are shown in Table 2. The SPA1 and SSI were normal distribution, but HCDA, HCR, PD, DAR2, and CBI were non-normal distribution.

**TABLE 2:** DCR parameters from Corvis ST.

Parameters	Mean ± SD [median (IQR)]	Range
HCDA (mm)	0.89 (0.09)	0.78–1.15
HCR (mm)	7.93 (1.43)	7.17–9.68
PD (mm)	5.05 (0.39)	4.89–5.80
DAR2	4.17 (0.76)	3.50–4.64
SPA1 (mmHg/mm)	107.27 ± 13.08	79.82–135.95
SSI	0.99 ± 0.13	0.73–1.27
CBI	0.01 (0.03)	0.00–0.17

### Anterior corneal surface displacement

**FIGURE 5:**
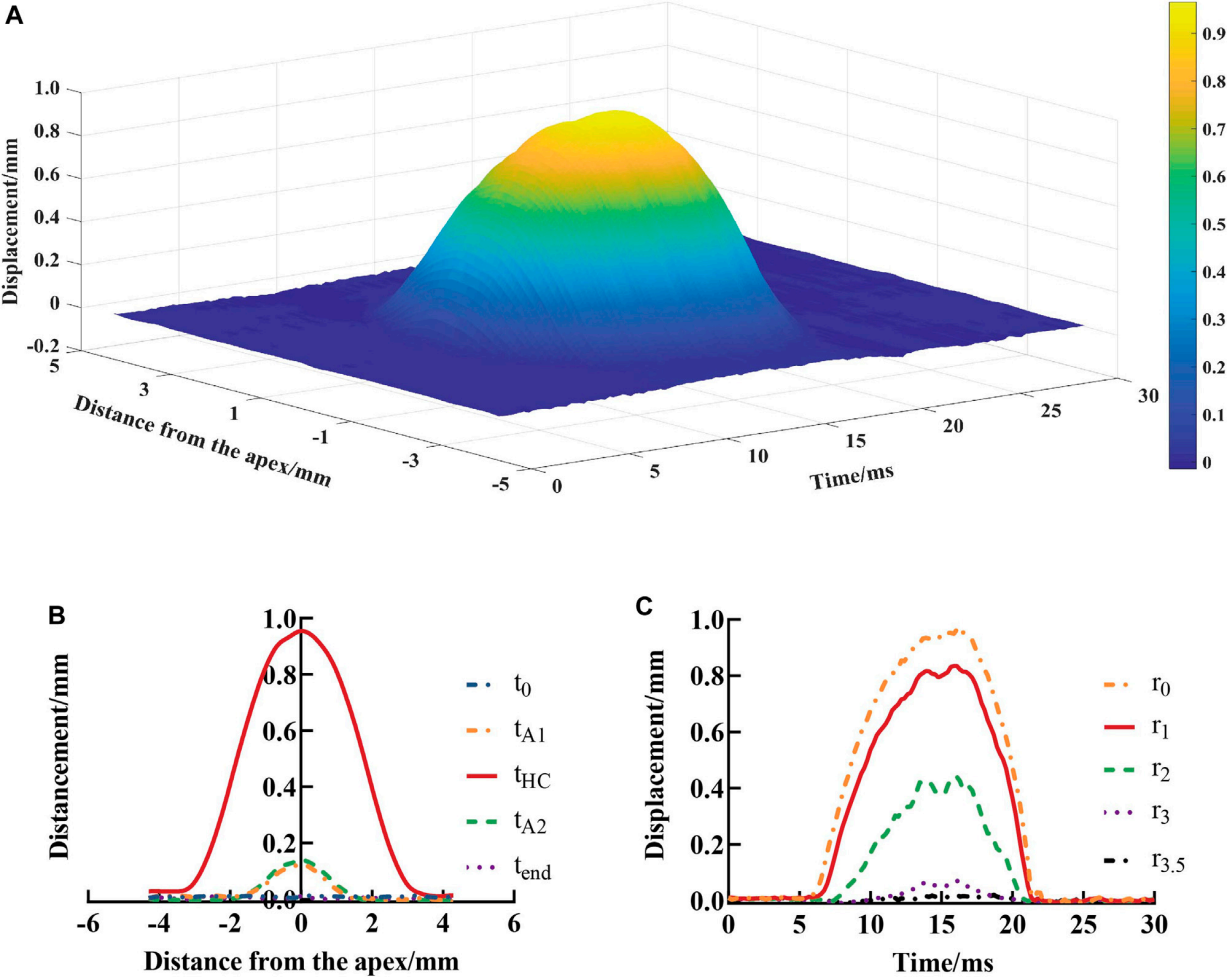
Vertical displacements of the anterior surface of the cornea at each position and test time point **(A)**, spatial distribution **(B)**, and temporal variation **(C)**. *t*_0_, *t*_A1_, *t*_HC_, *t*_A2_, and *t*_end_ represent the time at initial state, first applanation, highest concavity, second applanation, and end state, respectively; $r_{l}$ represents the distance from the corneal apex which was *l* (*l* = 0, 1, 2, 3, 3.5) mm.

For each subject’s 140-frame Corvis ST image data set, we extracted the contour data of the anterior corneal surface and then obtained the data set $\;\;\left(r_{j},\;t_{j},d_{ij}\right)$ , where i=−287, −286, ⋯, 286, 287;j=0, 1, ⋯, 139 , with $r_{-287}=r_{287}=4.29\;\text{mm},\;t_{0}=0,\;\;t_{139}=30\;\text{ms}$ , and $d_{ij}$ is the displacement of the anterior corneal surface at point (*i*, *j*). Figure 5 is the displacement data of the anterior corneal surface of a typical subject.

**FIGURE 6:**
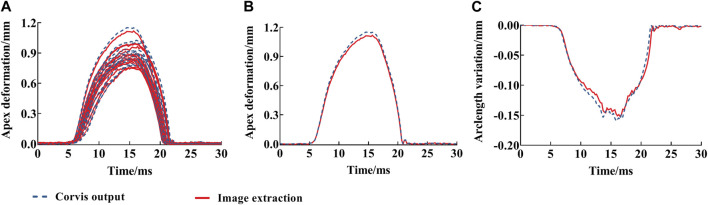
Deflection amplitude and arc length variation of corneal anterior surface vertices from image extractions with time: deflection amplitude of all eyes **(A)**, one eye **(B)**, and arc length variation **(C)**. The red solid line is the image extraction result; blue dotted line is the deflection amplitude from Corvis ST.

In order to verify the effectiveness of the displacement results from image extractions, the data set $\left(t_{j},\;d_{0j}\right)$ from image extractions was compared with the deflection amplitude (DA) output from Corvis ST. The root mean square (RMS) of displacement difference of about 140 data points was calculated. The RMS of displacement was 0.0245 ± 0.0092 mm (range: 0.0119–0.0439 mm). The corneal anterior surface vertex displacements from image extractions with time are shown in Figure 6A, which shows that the image extraction results gave a good agreement and closed to the DA with time. Moreover, the arc length variation data of the anterior corneal surface within 3.5 mm from the corneal apex with time was obtained from Corvis ST and was calculated as shown in Eq. 4 based on image extractions, respectively. Figure 6C is the comparison of arc length variation between the outputs of Corvis ST and then calculated for one typical eye. It only found slight differences (the RMS of arc length variation was 0.006 mm). Therefore, the accuracy of the data gotten from image extraction was comparable with that from Corvis ST.

*S*(*r*, *t*) (Eq. 6) was used to fit the vertical displacement of the anterior corneal surface at different distances from the corneal apex at different times, and the goodness of fit (*R* ^2^) was greater than 0.97 (Table 3).

**TABLE 3:** The fitted parameters of *S*(*r*, *t*).

Parameters	Mean ± SD [median (IQR)]	Range
*w*_0_	−0.0006 (0.0051)	−0.0573 to 0.0033
*w*_1_	−1.1220 (0.1378)	−1.2000 to −0.9283
*S*_1_	0.0109 (0.0092)	0.0000–0.0218
*S*_2_	0.0170 ± 0.0045	0.0100–0.0274
*S*_3_	0.0039 (0.0010)	0.0011–0.0078
*S*_4_	0.0006 (0.0002)	0.0001–0.0009
*a*_1_	0.4547 ± 0.1219	0.2304–0.6774
*a*_2_	0.6521 (0.1784)	0.5146–0.8000
*b*_1_	1.0341 ± 0.1189	0.8422–1.2000
*b*_2_	−0.6471 (0.3408)	−0.7599 to −0.6471
*c*_1_	1.2220 (0.2330)	0.9882–1.3180
*c*_2_	1.4755 ± 0.1323	1.2750–1.6920
*R* ^2^	0.9865 ± 0.0064	0.9742–0.9956

### Nonlinear Viscoelastic Parameters

Descriptive statistics of the model parameters identified based on the work done by air-puff force attaining the whole corneal displacement from Corvis ST are shown in Table 4. *μ* and *α* were normal distribution, and *A* and *τ* were non-normal distribution. Table 4 shows the median, mean, standard deviation, IQR, minimum, and maximum of the material parameters for all corneas of the participants, and the median of goodness of fit (*R* ^2^) was over 0.96. Furthermore, power of the tests calculated by using the data of *μ* was about 0.87.

**TABLE 4:** The determined material parameters.

Parameters	*μ* (MPa)	*α*	*A*	*τ* (ms)	*R* ^*2*^
Mean (median)	0.62	44.40	(0.23)	(1.35)	(0.969)
SD (IQR)	0.08	6.95	(0.16)	(0.35)	(0.025)
Range	0.42–0.74	32.76–55.63	0.09–0.36	1.21–1.95	0.951–0.988

**FIGURE 7:**
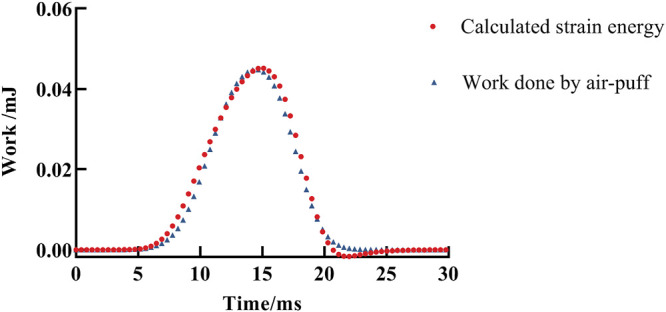
A fitting result of work done by air-puff force attaining the whole corneal displacement during the Corvis ST test by the visco-hyperelastic model. The red solid dot is the fitting result, and the blue triangle represents the calculated work done by air-puff force attaining the whole corneal displacement during the Corvis ST test.

A typical fitting result of the visco-hyperelastic model to the work–time curve for one eye is shown in Figure 7. It shows a good agreement and is close to the work done by air-puff force attaining the whole corneal displacement in the Corvis ST test with time.

## Discussion

This study established a method to identify the nonlinear viscoelastic properties of human corneas from the output data of Corvis ST. The results showed feasibility of the method.

The morphology of the normal cornea has almost no change under IOP. For abnormal cornea, corneal viscoelasticity plays an important role (Vellara and Patel, 2015) in the maintenance of corneal morphology under long-term IOP. Keratoconus, a common clinical disease characterized by progressive corneal dilation, is highly related to corneal viscoelasticity (Vellara and Patel, 2015; Jin et al., 2020; Rahmati et al., 2021). This study gives a more comprehensive understanding of corneal biomechanical properties from the test of cornea *in vivo*. Compared with other studies, the innovation of this research is the determination of the nonlinear viscoelastic properties of human corneas from the output data of Corvis ST. It is expected that the biomechanical properties of the human cornea will be widely used in the early diagnosis of keratoconus, the screening of cornea before refractive surgery, and the early diagnosis of iatrogenic corneal ectasia after refractive surgery.

IOP is always acting on the inner surface of the cornea. So the cornea has been under the tension generated by IOP, before, during, and after the Corvis ST test. In this study, the cornea under normal IOP has been regarded as an equilibrium state. It is similar in that the pre-stress is taken into account in corneal tests *in vitro*. For example, in the uniaxial tensile test of corneal strips, the stress–strain relationship should be $\text{σ}-\sigma_{0}=f\left(\varepsilon -\varepsilon_{0}\right)$ , where $\sigma_{0},\;\varepsilon_{0}\;$ are the initial stress and strain of the specimen, respectively. In many studies, the zero stress state has been taken as the initial state, i.e., $\sigma_{0}\;$ and $\varepsilon_{0}$ are zero (Elsheikh et al., 2010; Liu et al., 2020a; Liu et al., 2020b). According to the Laplace formula, the corneal stress under IOP is σ=pr/2t (*p*, *r*, and *t* are the IOP, corneal radius, and corneal thickness, respectively); if the IOP is 16 mmHg, corneal radius and corneal thickness are 6 and 0.57 mm, respectively, the tensile stress of the cornea is 0.0112 MPa under IOP. Therefore, this study regarded tensile stress of 0.0112 MPa as the equilibrium state of cornea, based on which the visco-hyperelasticity of the cornea was obtained. The identified material parameters may be called as *in vivo* corneal biomechanical parameters.

Our previous study suggested a method (Qin et al., 2019a), following the studies of Ko et al. (2013) and Reissner (1946), to determine the corneal elastic modulus from corneal apical displacements between 0.1 and 0.2 mm at the first applanation state during the Corvis ST test based on the relationship between force and displacements of the shallow spherical model. It gave a corneal elastic modulus of human which ranged from 0.16 to 0.30 MPa. Shih et al. (2017) took the average pressure of air-puff during the Corvis ST test as the external pressure, did not consider the pressure changes with time, and applied Taber’s model to obtain the elastic modulus of human which ranged from 0.053 to 0.363 MPa. These elastic moduli are given at the equilibrium of the first applanation state. The present study was initially motivated by Taber’s model (Shih et al., 2017; Taber, 1982) and applied a visco-hyperelastic model to determine corneal nonlinear viscoelastic parameters. According to Eq. 4 in the present study, the stretch ratio is about 0.995 at the first applanation state. This also demonstrates that our research was carried out under normal IOP as an equilibrium. Furthermore, in the studies of identifying corneal material parameters from Corvis ST data by the inverse finite element method, the zero stress state is also regarded as an initial state (Lago et al., 2015; Rahmati et al., 2021). Therefore, our study is different from most previous studies. We have given the *in vivo* corneal biomechanical parameters.

As we have known, the uniaxial stretch test is usually used to identify the tangential modulus of the cornea (Wollensak et al., 2003; Elsheikh et al., 2010; Zhang et al., 2018). If we let the stretch ratio $\lambda_{t}\;$ be from the tensile direction data and the stretch ratios from transverse and thickness directions be equal to each other, then from the Ogden model follows $U=\frac{2\mu }{\alpha^{2}}\left(\lambda_{t}^{\alpha }+2\lambda_{t}^{-\frac{\alpha }{2}}-3\right)$ . The tangential modulus of the cornea can be regarded as the second derivative of strain energy density function with respect to $\lambda_{t}$ , i.e., $E_{t}=\frac{2\mu }{\alpha }\left[\left(\alpha -1\right)\lambda_{t}^{\alpha -2}+\left(\frac{\alpha }{2}+1\right)\lambda_{t}^{-\alpha /2-2}\right]$ . According to the determined material parameters in the present study, the corneal tangential modulus is 1.61 ± 0.22 MPa when the strain is 0.01. Elsheikh et al. (2010) obtained the tangential modulus of the human cornea which was in the range of 0.32–1.66 MPa by uniaxial tensile test when the strain was about 0.01, and Liu et al. (2020b) obtained the elastic modulus of the corneal lenticule from young people which was about 1.2 MPa from the uniaxial tensile test when the strain is within 0.02. The tangential modulus at a strain of 0.01 obtained in this study may be compared with those at a strain of 0.02 or more provided by uniaxial tests. Since the elastic modulus that we calculated from Corvis ST data is under the normal IOP, this may result in slightly different results from previous studies.

The researchers believe that the strain rate under loading affects the stress–strain relationship of soft tissue (Snedeker et al., 2005; Kim et al., 2012) and that the stiffness of the cornea is directly proportional to its strain rate (Rahmati et al., 2021). It should be noted that this study is based on the test data of Corvis ST to identify the corneal nonlinear viscoelastic parameters. The test time is only about 30 ms, a fast loading and unloading process with the loading rate being about 2.5/s. This may lead to the difference between the parameters of the Ogden model obtained in this study and the results of the uniaxial tensile test of the *in vitro* cornea (strain rate less than 0.005/s). Moreover, the parameter *A* (0.10–0.39) is close to the values of *A*_k_ (*k* = 1, 2, 3) in Prony relaxation function, according to the studies by Kling et al. (2014) and Qin et al. (2019b). However, *τ* (1–2 ms) is smaller than *τ*_k_ (*k* = 1, 2, 3) in Prony relaxation function compared with the reported results. Because viscoelasticity is a time-dependent property, and the strain rate (∼2.5/s) during the Corvis ST test is larger than during the stress relaxation test (less than 0.1/s), this may lead to the smaller *τ* in the Prony series obtained in this study.

In addition, a visco-hyperelastic model has been used to describe the stress–strain curve of the kidney capsule obtained by the uniaxial tensile test at high strain rates, and to identify the biomechanical characteristics of the tissue (Snedeker et al., 2005); this indicates that the visco-hyperelastic model has the possibility to be applied to identify the biomechanical properties of soft tissue under rapid deformation. The results of our study also confirm this possibility. The established method provides the evaluation biomechanical properties of the human cornea *via in vivo* test.

In addition, there are some advantages and limitations of using the visco-hyperelastic model to describe the biomechanical behavior of the human cornea. First of all, visco-hyperelastic models based on strain energy functions have been shown to reproduce both the time-dependent and large strain aspects of the response (Ahearne et al., 2007; Boyce et al., 2007). A visco-hyperelastic model can give both elastic and viscous characteristics of the cornea. Secondly, this research showed that the calculation to determine model parameters is easy to carry out and saves time compared with inverse finite element methods. Thirdly, the simple model we selected, the convenient and fast computation, and the nonlinear viscoelasticity make the method of this study a potential method adopted by clinic applications, such as corneal screening before refractive surgery and early diagnosis of keratoconus. The limitation includes that researchers have not reached a consensus on how to identify the viscoelastic properties of the cornea based on Corvis ST data. The existing literature on whether the viscoelasticity of cornea can be evaluated from Corvis ST data implemented the study based on a specific constitutive model of cornea. Due to the different methods adopted, therefore, there were some differences in the results. Francis et al. (2019) believed that corneal viscous properties cannot be determined from air-puff applanation. However, majority of studies have shown that the viscoelasticity of the cornea can be obtained according to the output data of Corvis ST; for example, based on the output data of Corvis ST, the viscoelasticity of the cornea of normal people and patients with keratoconus has been compared and analyzed by the inverse finite element method (Rahmati et al., 2021), and Abass et al. (2020) believed that the viscoelasticity of the cornea can be determined by using the output data of Corvis ST. Our research showed that Corvis ST data can be used to explore the viscoelasticity of cornea. Further, Corvis ST records the morphological changes of cornea under the action of air-puff within about 30 ms, which is a rapid loading and unloading process. In the existing studies, the period of corneal stress relaxation tests was not less than 2 min (Hammer et al., 2015; Kling et al., 2015; Zhang et al., 2018). The difference in test time between Corvis ST and classical relaxation or creep tests may result in different orders of magnitude of the model parameters. How to understand the relationship between them and corneal stress relaxation characteristics needs to be further investigated.

There are some limitations in this study. First, the number of participants included was small, considering that the population with different cornea stiffness may have different responses to Corvis ST; its clinical significance needs to be further validated with study on patients with corneal biomechanics disorder, such as keratoconus, or patients that received corneal collagen crosslinking. Second, we studied the cornea with certain tension under IOP as the initial state and failed to obtain the biomechanical parameters of the cornea under zero stress state. However, we also note that the initial state that should be paid attention to in the research of applying corneal biomechanical properties to clinical problems is the state under IOP, so the method we provide to identify corneal biomechanical properties may be more useful. Third, in this study, we did not consider the corneal anisotropic mechanical properties. It should be further studied in the future.

## Conclusion

In conclusion, it is provided that the corneal nonlinear viscoelastic properties can be determined based on a visco-hyperelastic model from the corneal contour information and air-puff pressure of Corvis ST test. All in all, the present conclusion should be substantiated with more investigation, by which to provide more useful information for clinical practice.

## Data Availability

The original contributions presented in the study are included in the article/Supplementary Material, further inquiries can be directed to the corresponding authors.
